# Assessment of Emergency Medicine Residents’ Clinical Reasoning: Validation of a Script Concordance Test

**DOI:** 10.5811/westjem.2020.3.46035

**Published:** 2020-06-24

**Authors:** Eric Steinberg, Ethan Cowan, Michelle P. Lin, Anthony Sielicki, Steven Warrington

**Affiliations:** * St. Joseph’s University Medical Center, Department of Emergency Medicine, Paterson, New Jersey; † Mount Sinai Beth Israel, Icahn School of Medicine at Mount Sinai, Department of Emergency Medicine, New York, New York; ‡ Orange Park Medical Center, Department of Emergency Medicine, Orange Park, Florida

## Abstract

**Introduction:**

A primary aim of residency training is to develop competence in clinical reasoning. However, there are few instruments that can accurately, reliably, and efficiently assess residents’ clinical decision-making ability. This study aimed to externally validate the script concordance test in emergency medicine (SCT-EM), an assessment tool designed for this purpose.

**Methods:**

Using established methodology for the SCT-EM, we compared EM residents’ performance on the SCT-EM to an expert panel of emergency physicians at three urban academic centers. We performed adjusted pairwise t-tests to compare differences between all residents and attending physicians, as well as among resident postgraduate year (PGY) levels. We tested correlation between SCT-EM and Accreditation Council for Graduate Medical Education Milestone scores using Pearson’s correlation coefficients. Inter-item covariances for SCT items were calculated using Cronbach’s alpha statistic.

**Results:**

The SCT-EM was administered to 68 residents and 13 attendings. There was a significant difference in mean scores among all groups (mean + standard deviation: PGY-1 59 + 7; PGY-2 62 + 6; PGY-3 60 + 8; PGY-4 61 + 8; 73 + 8 for attendings, p < 0.01). Post hoc pairwise comparisons demonstrated that significant difference in mean scores only occurred between each PGY level and the attendings (p < 0.01 for PGY-1 to PGY-4 vs attending group). Performance on the SCT-EM and EM Milestones was not significantly correlated (r = 0.12, p = 0.35). Internal reliability of the exam was determined using Cronbach’s alpha, which was 0.67 for all examinees, and 0.89 in the expert-only group.

**Conclusion:**

The SCT-EM has limited utility in reliably assessing clinical reasoning among EM residents. Although the SCT-EM was able to differentiate clinical reasoning ability between residents and expert faculty, it did not between PGY levels, or correlate with Milestones scores. Furthermore, several limitations threaten the validity of the SCT-EM, suggesting further study is needed in more diverse settings.

## INTRODUCTION

A primary goal of residency training is to develop competence in clinical reasoning; however, there are no instruments that can accurately, reliably, and efficiently assess clinical decision-making ability. Current methods of clinical reasoning assessment such as simulation, written tests, clinical shift evaluations, and standardized patient encounters lack the optimal combination of fidelity (emulates real life), feasibility (easily reproduced), and content validity (evidence that the assessment measuring what it is intended to measure). Multiple-choice exams force learners to select single, predetermined “correct” answers, but fail to capture the uncertainty surrounding clinical scenarios.^1^ Essay-based examinations are time-intensive and have poor evaluator inter-rater reliability.^2^

Simulated clinical scenarios are an excellent means of assessing clinical reasoning skills; and due to their high-fidelity nature, they may assess a more realistic level of competence. However, simulation sessions cannot offer a wide array of clinical scenarios during a brief encounter due to the extensive need for time and resources. A single simulation session for 30 residents may take 15 hours of preparation time for faculty and technicians.^3^ Standardized patient encounters allow for assessment of clinical reasoning in a more realistic setting but are resource-intensive and time consuming.^4^ Finally, the frequently used end-of-shift evaluations are subject to bias, may be subjective, and may result in grade inflation.^5^

The script concordance test (SCT) is designed to measure clinical reasoning ability in the context of uncertainty.^6^ The advantages of the SCT in comparison to the aforementioned strategies are that it is more congruent with actual clinical practice in emergency medicine (EM), in which decisions are often made in the face of ambiguity. In addition, the SCT has the ability to assess examinees’ responses to several clinical scenarios yet is easy to administer and score.^7^ The SCT accomplishes these tasks by presenting the trainee with multiple clinical scenarios and comparing their responses to an expert panel, rather than selecting one correct option. In 2011, Humbert et al developed and assessed a SCT in EM (SCT-EM), which evaluated clinical reasoning skills among EM residents and medical students.^7^ The SCT-EM was able to discriminate among examinees with varying levels of clinical experience (ie, medical students vs residents vs experts). To establish convergent validity, the authors compared the SCT-EM to the American Board of Emergency Medicine (ABEM) in-training exam and the United States Medical Licensing Examination (USMLE) Step 2-Clinical Knowledge (CK) exam. However, the in-training exam only measures one dimension of clinical reasoning (knowledge), while the Step 2-CK exam is not specific to EM and is typically completed before residency training. Furthermore, it is unknown whether the SCT-EM can be used to measure an EM resident’s progression during training.

We aimed to expand upon evidence supporting the validity of the SCT-EM by determining whether it could reliably distinguish clinical reasoning ability between EM residents by postgraduate year (PGY) level. We also attempted to validate Humbert’s SCT instrument by comparing SCT-EM results to the EM Milestones, a method endorsed by the Accreditation Council for Graduate Medical Education (ACGME) to assess and benchmark clinical competency.^8^–^10^

**Population Health Research Capsule**

What do we already know about this issue?

*Assessing clinical reasoning in emergency medicine (EM) residents is difficult. Many methods of doing so exist, each with its own pros and cons*.

What was the research question?

Is a script concordance test an accurate and reliable tool to assess EM residents’ clinical reasoning skills?

What was the major finding of the study?

*The script concordance test for EM has limited utility in reliably assessing clinical reasoning among EM residents*.

How does this improve population health?

*By assessing the crucial skillset of clinical reasoning during residency, the ability for future emergency physicians to effectively manage patients may be improved*.

## METHODS

### Study Design

We performed a cross-sectional study of EM residents comparing SCT-EM scores among and between EM residents of different PGY years and expert attending emergency physicians. We then correlated EM residents’ SCT-EM scores to their subsequent ACGME “Patient Care” Milestones scores 1–6, which focus on emergency stabilization, diagnostic studies, diagnosis, pharmacotherapy, and observation and reassessment, respectively.

### Study Setting and Population

We enrolled EM trainees and board-certified attending faculty physicians (“experts”) in three residency programs (two PGY 1–3 format, one PGY 1–4 format) in an urban academic setting. While the three residency programs evaluated were all part of a single health system, each program had distinct faculty, clinical sites, and conference structures.

### Study Protocol

The SCT-EM is a 59-question assessment consisting of 12 clinical vignettes typically encountered in the emergency department, originally developed by two test-writers (AJH and BB) in Humbert et al 2011.^7^ The questions were categorized as diagnostic, investigational, or therapeutic. Based on previous evidence on how to optimally construct a SCT, Likert-scale response choices were attached to each question.^11^,^12^ For example, take a hypothetical patient who presents with a chief complaint of headache. The clinical decision-making process (i.e., what differential diagnoses to entertain, what studies to order, what therapeutic options to consider) is dependent on information obtained from the history, physical exam, and investigational studies. The SCT-EM is developed such that elements from the history and physical exam as well as investigational studies are introduced to the examinee in the context of a clinical vignette; and this new information may or may not be useful in his or her clinical decision-making process. Respondents indicate via a five-point Likert scale (−2, −1, 0, 1, 2), the degree of effect that a new piece of information has on the clinical decision they are to make. An example of an SCT-EM item is provided in Appendix A.

As eight years have elapsed since the original SCT-EM exam was developed, two reviewers (ES and EC) examined the SCT-EM scenarios to assess face validity, that is, to ensure that there were no major changes in diagnostic, investigational, or therapeutic principles regarding the test items. Neither reviewer believed that any of the questions required alteration or removal. As per the original study protocol, a scoring key was derived by administering the examination to an expert panel consisting of board-certified EM faculty from all three residency training sites.

Residents were recruited on a voluntary basis to take the exam during a weekly educational conference in November 2018. Instead of expanding the enrollment period to collect more responses, we deliberately recruited in this very narrow timeframe to minimize variability in residency experience between the subjects of the same PGY year. After obtaining verbal consent by a co-investigator who did not have a leadership role within the residency program, the test was administered with paper and pencil. Residents and members of the expert panel were given 45 minutes to complete the examination. Upon completion of the exam, examinees voluntarily completed a brief survey assessing their attitudes toward the SCT-EM. The study was reviewed and approved by a single institutional review board that reviews research for the health system and medical school.

### Data Collection

To score the SCT-EM, one full credit (one point) was awarded to a response that correlated to the modal answer provided by the expert panel. Partial credit was also obtainable on the SCT-EM, by calculating the ratio of congruent expert responses to that of the modal response. For example, of a 10-person expert panel, if eight answered “0” and two answered “−2” for a particular item, those examinees with the modal response, “0,” would receive one full point, those who responded “−2” would receive 0.2 (2/10 experts with the same answer), and all other responses would receive no credit. An example of our scoring matrix is available in Appendix B.

ACGME EM Milestones “Patient Care (PC)” competency scores were obtained from the Fall 2018 clinical competency committees’ semi-annual meetings from each residency training program. Data were recorded in an electronic database by a co-investigator blinded to the study outcomes using Microsoft Excel (Microsoft Corp., Redmond, WA). SCT-EM responses were de-identified by assigning each participant a unique code to ensure participant confidentiality. Once SCT-EM scores had been matched to the Milestone scores, all identifying information was removed.

### Data Analysis

We analyzed baseline characteristics of the groups using descriptive statistics. Mean and standard deviations were calculated for normally distributed continuous variables and proportions for categorical variables. We analyzed normality of SCT scores and Milestones using Shapiro-Wilk normality tests and graphical methods. Mean SCT scores were compared using pairwise comparison of means. Tukey’s procedure was used to adjustment for multiple comparisons. The alpha level was set at 0.05.

We performed correlation between SCT and milestone scores by calculating Pearson’s correlation coefficients. Simple linear regression was used to produce a fitted correlation line and R-squared value to overlay onto a scatterplot comparing SCT exam scores to Milestone scores. Inter-item covariances for SCT items were calculated using Cronbach’s alpha statistic. Sample size was based on the total pool of eligible residents in the three surveyed residency programs. We analyzed all statistical data using Stata, Version 15.1 (StataCorp, College Station, TX).

## RESULTS

### Population and Program Characteristics

Of 138 eligible residents from three different EM residency programs, a total of 68 (49%) completed the SCT-EM. One resident did not indicate a PGY year and was not included in the final analysis. Of the residents completing the SCT, 22 (32%) were PGY-1s, 21 (31%) were PGY-2s, 19 (28%) were PGY-3s, and six (9%) were PGY-4s. For the two PGY 1–3 programs 62% and 51% of residents completed the SCT-EM, respectively. For the one PGY 1–4 program 46% of residents completed the SCT-EM. Of the 15 attending physicians from three different programs asked to compile the expert panel, 13 (87%) completed the SCT-EM. Each member of the expert panel completed all 59 questions.

### Script Concordance Test-EM Scores

Mean SCT scores for each group are shown in Table 1. Mean differences in SCT scores between all groups (PGY-1, 2, 3, 4, attending) was statistically significant (p<0.001). Post hoc pairwise testing demonstrated no significant difference in SCT scores between PGY groups. The difference between SCT scores between the attending group and all PGY groups except the PGY-4 group was statistically significant (P< 0.001, Table 2).

### Milestones Scores and Convergent Validity

There was no correlation between performance on the SCT exam and Milestone scores (r = 0.12, p = 0.35), as demonstrated on the Figure.

### Test Performance

The Cronbach’s alpha for correlation of SCT scores among all test takers was 0.68 (n = 81). Among the panel of experts, the alpha increased to 0.89.

### Survey Results

Of the 65 respondents, 45 (73%) agreed that the test was easy to understand; 57/61 (93%) respondents felt that there was enough time to complete the test; and 56/62 (90%) agreed that the clinical scenarios were realistic.

## DISCUSSION

This is the first study to stratify SCT-EM scores among EM residents by PGY year, as well as the first to compare SCT-EM scores to ACGME Milestones scores. While the SCT-EM did not differ between PGY levels, the exam was able to differentiate clinical reasoning skills between residents and expert physicians. This confirms prior study results across various specialties.^7^, ^13^–^31^ Considering the expert panel achieved significantly higher scores than all resident groups, yet there was no significant difference between resident groups, our findings raise the possibility of an inflection point of clinical reasoning ability that occurs sometime between graduating residency and practicing independently. Literature suggests that more experienced emergency physicians “differ from novices in clinical decision-making strategy by their ability to focus and be selective.”^32^ In addition, it has been suggested that expert physicians take advantage of their accumulated knowledge and experiences to make clinical decisions in a more purposeful manner.^33^

In terms of assessing convergent validity, performance on the SCT-EM did not correlate with ACGME Milestones scores, a universally accepted framework of assessment. Specifically, we chose sub-competencies “PC 1–6,” which focuses on patient care and clinical decision-making. This raises the concern that ACGME Milestones scores may not be associated with clinical reasoning ability, or that the SCT-EM may measure another important aspect of clinical reasoning assessment that is not encompassed by Milestones. Humbert et al noted a modest positive correlation between SCT-EM scores and USMLE Step 2-CK performance, establishing convergent validity.^7^ Higher performance on the USMLE Step 2-CK may predict higher first-time pass rates on oral board examinations, and ABEM qualifying exams.^34^,^35^ Further research is needed to establish the association between written board examination scores and clinical reasoning ability and/or quality of patient care.

Our study also establishes the feasibility and acceptability of administering the SCT-EM. A majority of the participations agreed that the test was easy to understand, that there was enough time to complete the test, and that the scenarios were realistic. These findings comport with prior studies that SCTs are easy to administer and represent clinical situations that translate into real practice.

Although SCTs are typically regarded as an assessment tool, there is great potential for their use as a unique instructional modality.^7^ The SCT-EM could be used to facilitate a scenario-based dialogue between residents and an expert panel of attendings, justifying and challenging each other’s rationales behind their thought processes and decisions. These discussions could add valuable qualitative information to a quantitative exam. One prior study applied a “think aloud” approach in which examinees reflected upon their reasoning in written form as they completed a SCT. The authors found that this strategy enhanced the examinees’ ability to critically evaluate their own clinical reasoning skills compared to interpreting their SCT results alone.^36^ Another study in which an SCT was used for a continuing medical education curriculum found high rates of learner satisfaction and self-assessed knowledge acquisition and change in practice.^37^ Further research is needed to evaluate the SCT as an instructional strategy for resident education.

## LIMITATIONS

While our study was limited by the convenience sample and response rate, all PGY levels were well represented. This 49% response rate may instill a substantial risk of responder bias. The lack of a difference in mean SCT-EM scores between PGY years may be due to sample size, as a power analysis was not performed to determine the sample size necessary to produce statistically significant results. Our findings may have limited generalizability because it was conducted in three urban residency programs in close proximity to each other, under one GME hospital system. However, the three programs represent a broad range of clinical settings, including community and academic.

We assessed convergent validity using the residents’ ACGME Milestones scores; however, ACGME Milestones scores have been suggested to lack inter-rater reliability, and consistency between GME programs.^38^–^39^ Moreover, the ACGME Milestones are not a complete determination of residents’ abilities nor do they assess all areas essential to unsupervised practice.^40^ Finally, the ACGME Milestones were not designed to be an assessment tool in this context. Despite these limitations, Milestone scores are endorsed by the ACGME to assess and benchmark clinical competency and used by all EM residency programs.

The SCT itself may have several implicit weaknesses. For one, respondents may perform significantly better on the exam by avoiding extreme responses (i.e., −2 or 2).^41^,^42^ Secondly, critics posit that SCT reliability evidence essentially ignores inter-panelist and test-retest measurement error by simply using levels of coefficient alpha as a surrogate for reliability.^42^ Next, it is impossible to determine whether an examinee has an awareness of divided expert opinion or probability beliefs regarding cases prior to the exam.^42^ In addition, the face validity of the SCT may be dependent on the quality of the exam questions, particularly the amount of context offered in each clinical vignette.^43^ Finally, our study was based on an assumption that clinical reasoning could be adequately measured using one assessment tool. Young et al highlight the extent of this misconception, stating that standardized tests may not properly capture how well trainees perform in setting of uncertainty.^44^ Considering these limitations, the utility of the SCT-EM may lie with formative assessment rather than high-stakes evaluation.

## CONCLUSION

Clinical reasoning ability is difficult to reliably and feasibly assess. Although our findings demonstrate that the SCT-EM had ability to differentiate clinical reasoning ability between residents and expert faculty, it was unable to differentiate clinical reasoning between PGY levels. There are several proposed limitations inherent to the script concordance test, calling into question its overall ability to assess clinical reasoning. Future studies examining differences among residents as they progress during and after residency training, or in different residency settings, may elucidate the utility of the SCT-EM.

## Supplementary Information





## Figures and Tables

**Figure:**
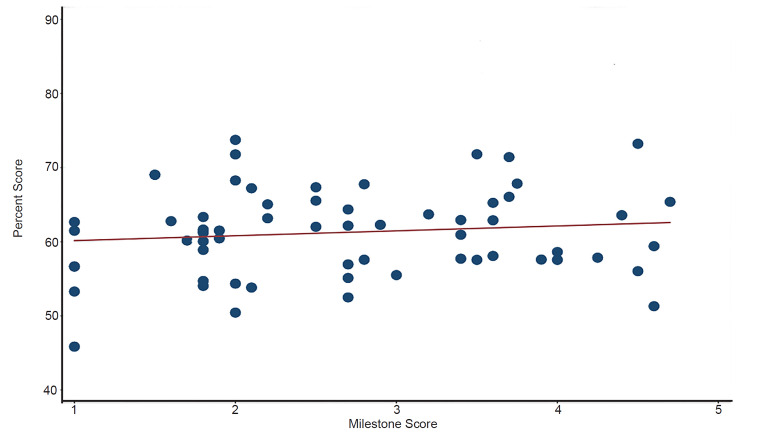
Scatterplot showing a fitted regression line comparing Accreditation Council of Graduate Medical Education Milestone scores to script concordance test (SCT) scores. r= 0.12, p= 0.35, R-squared= 0.01. *SCT*, Script Concordance Test.

**Table 1:** Descriptive statistics by group.

Group	Mean (%)	SD	Sample Size	Range (%)
PGY-1	58.5	4.1	22	44.7–69
PGY-2	62.2	3.5	21	52.5–73.7
PGY-3	60.5	4.1	19	43.1–71.8
PGY-4	61.5	4.5	6	51.3–73.2
Experts	72.8	4.9	13	57.5–85.7

*PGY*, postgraduate year; *SD*, standard deviation.

**Table 2:** Post-hoc testing: pairwise mean comparisons demonstrating mean differences in script concordance test scores between groups.

Group	Mean Difference	Standard Error	Tukey 95% CI
PGY-2 vs PGY-1	2.1	1.3	−1.4 to 5.5
PGY-3 vs PGY-1	1.1	1.3	−2.5 to 4.7
PGY-4 vs PGY-1	1.7	1.9	−3.6 to 7.0
Attending vs PGY-1*	8.4	1.4	4.4 to 12.4
PGY-3 vs PGY-2	−0.9	1.3	−4.6 to 2.7
PGY-4 vs PGY-2	−0.3	1.9	−5.6 to 5.0
Attending vs PGY-2*	6.3	1.5	2.3 to 10.4
PGY-4 vs PGY-3	0.6	1.9	−4.5 to 6.0
Attending vs PGY-3*	7.3	1.5	3.1 to 11.4
Attending vs PGY-4	6.6	2.0	1.0 to 12.3

* indicates p <0.001.

*PGY*, postgraduate year; *CI*, confidence interval.
