# Supplementary material for: Assessment of Emergency Medicine Residents’ Clinical Reasoning: Validation of a Script Concordance Test
Source: West J Emerg Med. 2020 Jun 24;21(4):978–84. doi: 10.5811/westjem.2020.3.46035 (PMC7390545; doi:10.5811/westjem.2020.3.46035)
Supplement: Supplementary file 1 [file wjem-21-978-s001.docx]

**Appendix A.** Sample SCT-EM Items. From “Assessing Clinical Reasoning Skills in Scenarios of Uncertainty: Convergent Validity for a Script Concordance Test in an Emergency Medicine Clerkship and Residency,” by A. J. Humbert et al. 2011. Copyright 2011 by A. J. Humbert. Reprinted with permission.

**
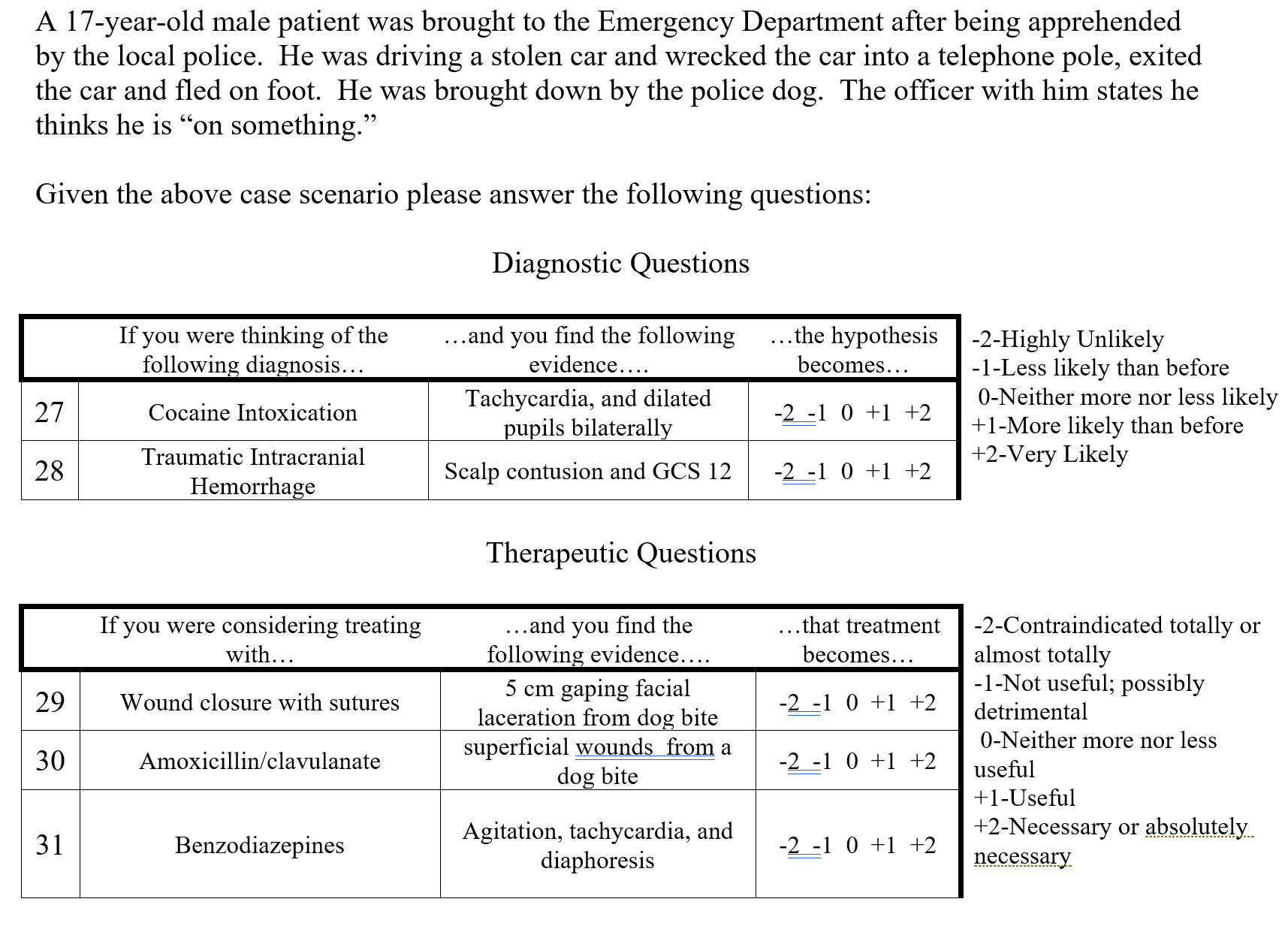
**
