# Supplementary material for: Assessment of Emergency Medicine Residents’ Clinical Reasoning: Validation of a Script Concordance Test
Source: West J Emerg Med. 2020 Jun 24;21(4):978–84. doi: 10.5811/westjem.2020.3.46035 (PMC7390545; doi:10.5811/westjem.2020.3.46035)
Supplement: Supplementary file 2 [file wjem-21-978-s002.docx]

**Appendix B.** Sample tally of expert responses and derivation of scoring matrix.

| **Expert Panel Response Tally** | | |  |  | |  |
| --- | --- | --- | --- | --- | --- | --- |
|  |  |  |  |  | |  |
|  | **Q5** | **Q6** | **Q7** | **Q8** | | **Q9** |
| **-2** | 0 | 1 | 0 | 0 | | 0 |
| **-1** | 6 | 3 | 0 | 0 | | 0 |
| **0** | 7 | 9 | 1 | 7 | | 0 |
| **1** | 0 | 0 | 5 | 0 | | 10 |
| **2** | 0 | 0 | 7 | 6 | | 3 |
|  |  |  |  |  | |  |
| **Scoring Matrix** | |  |  |  | |  |
|  | **Q5** | **Q6** | **Q7** | | **Q8** | **Q9** |
| **-2** | 0 | 0.08 (1/13) | 0 | 0 | | 0 |
| **-1** | 0.46 (6/13) | 0.23 (3/13) | 0 | 0 | | 0 |
| **0** | 1 (modal) | 1 (modal) | 0.08(1/13) | 1 (modal) | | 0 |
| **1** | 0 | 0 | 0.38 (5/13) | 0 | | 1 (modal) |
| **2** | 0 | 0 | 1 (modal) | 0.46 (6/13) | | 0.23 (3/13) |
